# A Time-Dependent Quantum Approach to Allostery and a Comparison With Light-Harvesting in Photosynthetic Phenomenon

**DOI:** 10.3389/fmolb.2020.00156

**Published:** 2020-08-28

**Authors:** Giovanni Villani

**Affiliations:** Istituto di Chimica dei Composti OrganoMetallici (UOS Pisa) – CNR, Area della Ricerca di Pisa, Pisa, Italy

**Keywords:** allosteric effect, quantum approach, time-depend, model of allostery, perturbation wave

## Abstract

The allosteric effect is one of the most important processes in regulating the function of proteins, and the elucidation of this phenomenon plays a significant role in understanding emergent behaviors in biological regulation. In this process, a perturbation, generated by a ligand in a part of the macromolecule (the allosteric site), moves along this system and reaches a specific (active) site, dozens of Ångströms away, with a great efficiency. The dynamics of this perturbation in the macromolecule can model precisely the allosteric process. In this article, we will be studying the general characteristics of allostery, using a time-dependent quantum approach to obtain rules that apply to this kind of process. Considering the perturbation as a wave that moves within the molecular system, we will characterize the allosteric process with three of the properties of this wave in the active site: (1) t_a_, the characteristic time for reaching that site, (2) A_a_, the amplitude of the wave in this site, and (3) B_a_, its corresponding spectral broadening. These three parameters, together with the process mechanism and the perturbation efficiency in the process, can describe the phenomenon. One of the main purposes of this paper is to link the parameters t_a_, A_a_, and B_a_ and the perturbation efficiency to the characteristics of the system. There is another fundamental process for life that has some characteristics similar to allostery: the light-harvesting (LH) process in photosynthesis. Here, as in allostery, two distant macromolecular sites are involved—two sites dozens of Ångströms away. In both processes, it is particularly important that the perturbation is distributed efficiently without dissipating in the infinite degrees of freedom within the macromolecule. The importance of considering quantum effects in the LH process is well documented in literature, and the quantum coherences are experimentally proven by time-dependent spectroscopic techniques. Given the existing similarities between these two processes in macromolecules, in this work, we suggest using Quantum Mechanics (QM) to study allostery.

## Introduction

The emergence of life in a system includes so wide a range of aspects and meanings to prevent us from giving reductive and univocal definitions of “life.” There are, of course, many definitions of the concept of life; each of them accentuates important aspects of life and highlights processes, and/or cycles of processes, that are indispensable to life, at least in the forms and ways we know it on Earth. Here we will not deal with these general issues. This paper focuses on the emergence of life in the cell. We believe that the importance of regulating the fundamental processes of life is an essential component, right from its definition. The allosteric effect is one of the most important processes for regulating the function of macromolecules (of proteins, in particular). The elucidation of the general characteristics of this phenomenon plays a major role in understanding the emergence of cellular life.

Allostery has been defined as the phenomenon in which a ligand, bound to one site (allosteric site or sensor) of a protein, alters the binding interaction of the protein with a second ligand in another site (active site). Here, we prefer to use a more general definition of this phenomenon. Nowadays, allostery is generally defined as any process were an event in a macromolecule site affects functions, dynamics, or distribution of the conformations of another site.

Allostery is a remote regulation through the transmission of information from one site to another, and this process is strictly related to the intramolecular dynamics in proteins. Allosteric effectors communicate with the active site by altering protein dynamics either through large-scale structural changes or through more subtle changes in correlated residue motions. A proper understanding of the allosteric mechanism is impossible unless the underlying dynamic conformational flexibility and energetic landscape are fully appreciated.

Schematizing a protein with an internal network of interactions of the components, the study of the allosteric process allows us to understand how an environmental perturbation, exerted in a part of the macromolecule, can move inside it and reach a specific distant site. In this sense, allostery becomes a characteristic of all macromolecules that are considered part of a complex interaction network among the component elements. The interactions in these networks allow the formation of “channels” within these systems, and, along these channels, a perturbation, generated in a specific site, can move. At this point, due to the presence of countless interactions and channels in the macromolecule, the problem is not to explain why the perturbation moves in the network but to explain how it is possible to avoid the complete dissolution of the perturbation. Moreover, the problem of the quantitative efficiency of this phenomenon arises because allostery is an action-at-a-distance force, and the active site can be many Ångströms away from the sensor site (Nussinov, 2016) [the average distance is 30–40 Å (Changeux and Edelstein, 2005)].

In an even more general sense, what will be said in this article for the allosteric phenomenon in a macromolecule is also valid for self-assembled systems in which smaller molecules are connected by weak interactions to form larger systems. In fact, the approach used here does not have the limitation of only being applicable to a macromolecule; even a super-system, where weak bonds connect the components (molecules or even fragments of macromolecules), can be treated in this way. We can study these super-systems in terms of a complex network of sites with interactions between them. Even in these cases, there is the general problem of understanding how a perturbation, located in a point within this network, can move and efficiently reach a distant site without completely dissolving.

Another fundamental process for life that shares some characteristics with allostery is the light-harvesting process (Jang and Mennucci, 2018) in photosynthesis. This phenomenon can be generally schematized in three distinctive processes: light-harvesting (LH), charge separation, and biochemical reaction. Here we will deal only with the first process: a quantity of energy arrives to the macromolecular antenna site to be later transferred to the reaction site, where it will be transformed into charge separation chemical energy.

The similarity between allostery and the LH process is that in both processes the two sites are dozens of Ångströms away; additionally, it is important in both cases that the initial energy is transferred efficiently without dissipating in the infinite degrees of freedom within the macromolecule.

The LH process has been extensively studied, and a certain number of coupled sites, which allow the propagation of the perturbation, besides the start site and the arrival site, have been identified. The importance of taking into account the quantum effects in this process has been well documented (Jang and Mennucci, 2018). The presence of quantum coherences, in fact, have been well proven by spectroscopic time-dependent techniques. This paper suggests that methods applied to the LH process can be used for allostery too, and the need for a quantum approach to allostery can be justified.

Undoubtedly, the most powerful experimental technique used to investigate the structural changes of macromolecules is NMR spectroscopy. By utilizing these techniques, a wide variety of parameters can be measured and reported such as the structure and dynamics of biomolecules over a wide range of time scales. The biggest advantage of NMR, compared to other techniques, is that it provides structural restraints for hundreds to thousands of individual atoms, or small groups of atoms, per sample per experiment (Clore, 2008). Due to its high resolution and inherent ability to capture structures and dynamics that underlie allosteric couplings, NMR spectroscopy has played a critical role in delineating the allosteric behavior (Kern and Zuiderweg, 2003; Kalodimos, 2011; Tzeng and Kalodimos, 2011; Manley and Loria, 2012; Masterson et al., 2012; Manley et al., 2013; Boulton and Melacini, 2016). NMR provides information for atomic resolution; however, increased spectral complexities, chemical shift overlaps, and short transverse relaxation times present problems in the application of NMR methods to large systems. At the same time, X-ray scattering, which is not limited by molecular weight, only provides information regarding the overall shape and size of the system. In such cases, hybrid methods that couple sparse NMR data with structural information from other lower-resolution techniques, such as cryo-electron microscopy (Loquet et al., 2012) or small/wide-angle X-ray scattering (SAXS/WAXS) (Schwieters et al., 2010; Takayama et al., 2011) have been shown to be very powerful in solving the structure of complex molecular systems. Moreover, rigid-body calculations, combining structures of NMR with low X-ray scattering data, can provide a powerful approach for studies of conformational dynamics in large proteins (Venditti et al., 2016).

From a theoretical point of view, the allosteric process has been mainly studied alongside thermodynamics. This approach allows modeling of the energetic aspects of the system but it does not allow the mechanism of the process to be derived. The study of the mechanism of this process has been tackled with classical mechanics (CM). Molecular dynamics (MD) simulation studies in CM have been applied to this process with algorithms that predict both allosteric sites and the mechanism of their connection with the active sites. The most straightforward way for studying allostery in MD is to run the simulations of both the apo-structure (the structure of the protein without the bond with the ligand) and the effector-bound protein structure and identify the main variations of the trajectories by comparing these two cases. In any case, since the routine molecular simulation datasets now comprise of terabytes of data, the direct visualization of raw MD trajectories is not possible. Therefore, the primary use of MD trajectories is in parameterizing a quantitative model of the structure of the system of interest. In particular, the main use of simulations with CM is for identifying allosteric networks, i.e., clusters of linked residues that propagate allosteric signals. Several algorithms (Kannan and Vishveshwara, 1999; Ghosh and Vishveshwara, 2007; Sethi et al., 2009; Gerek and Ozkan, 2011; Van Wart et al., 2014) have been developed for this use, starting from the original algorithm of Kannan and Vishveshwara (1999) in which residues are linked into clusters when tertiary contacts between side chains persist during a simulation. To demonstrate that the allosteric pathways identified in this way are robust, a community network analysis was carried out [introduced by Sethi et al. (2009)].

It is well known that while simulations can accurately predict many important molecular motions, these simulations are poorly suited to systems were quantum effects are important and they cannot be modeled with a traditional force field. The presence of transition metal atoms, an important transfer of protons, or a breakdown and/or bond formation are examples of these effects. Even when there is no break or formation of bond, the simulations generally ignore, with a few exceptions, the electronic polarization, caused by the flow of electrons from one atomic nucleus to another, in a quantum-mechanical effect. In classical MD simulations, we assign to each atom a fixed partial charge before the simulation begins. However, the electron clouds surrounding the atoms move constantly according to their environments, so that it would be better to consider the partial charges of the atoms as variable during the simulation. Despite wide agreement on the importance of accounting for electronic polarization, after 30 years of development a generally accepted polarizable force field has not been forthcoming.

In any case, the study by these simulation-based methods in CM approach often facilitate allosteric analysis of a protein (Ghosh and Vishveshwara, 2007; Sethi et al., 2009; Gasper et al., 2012; Van Wart et al., 2012; Collier and Ortiz, 2013; Freddolino et al., 2013; Yan et al., 2014; Wagner et al., 2016). In general, the atomic forces that govern molecular movement in these simulations are divided into two parts: those caused by interactions between bonded atoms and those caused by interactions between non-bonded atoms. Chemical bonds and atomic angles are included in the simple spring model, and the rotations around a bond are modeled with a sinusoidal function that approximates the energy differences between eclipsed and staggered conformations for the dihedral angles. Non-bonded forces due to van der Waals interactions are modeled using the Lennard-Jones potential, and electrostatic interactions are modeled using Coulomb’s law. The forces on each atom are then used to update the atomic positions at each step according to Newton’s laws of motion. Simulation time is typically discretized into steps of 1 or 2 fs, as required to accurately simulate the fastest atomic motions (i.e., hydrogen bond stretching), and the process is repeated many times. In order to reproduce the actual behavior of real molecules in motion, the energy terms described above are parameterized to fit quantum-mechanical calculations and experimental (for example, spectroscopic) data. This parameterization includes identifying the ideal stiffness and lengths of the springs that describe chemical bonding and atomic angles, determining the best partial atomic charges used for calculating electrostatic-interaction energies, and identifying the proper van der Waals atomic radii. Collectively, these parameters are called a “force field” because they describe the contributions of the various atomic forces that govern MD. Several force fields are commonly used in MD simulations, including AMBER (Cornell et al., 1995; Wang et al., 2004), CHARMM (Brooks et al., 1983), and GROMOS (Christen et al., 2005).

Quantum mechanics (QM) can give a significant contribution to the study of this phenomenon in three ways. The first use of QM in the study of allostery, as we said, is to determine which force field to use in the MD simulations. The second can be a detailed study of some specific residues considered essential in the allosteric phenomenon of a system. This can be done after modeling the system by the help of experimental data and/or theoretical simulations and the identification of few residues that can be used to schematize the main structures involved in the allosteric phenomenon. However, an accurate quantum calculation can only be done for smaller residues. This means a system must have hundreds, if not thousands, of atoms (using the geometry optimization) to create an accurate calculation (if this step is not necessary, we have, in a different context, performed an accurate quantum mechanical calculation of a system with 843 atoms (Villani, 2017, 2018) but without the optimization of the structure studied). Moreover, this kind of quantum study does not allow an easy generalization of the specific case nor does it allow general considerations on the allosteric process to be attained. Finally, another use of QM in the study of allostery could be to create general modeling rules for this process. In this paper, we will study allostery with this final use in mind. In fact, in spite of notable successes in studying allostery in specific systems, we have achieved much less success in identifying a set of general rules that provide an understanding of how allostery works.

One of the most important problems with a time-dependent approach to allostery is understanding why a perturbation, generated in a part of a macromolecule and free to move, does not propagate everywhere, diluting itself in the innumerable degrees of freedom within the system. In fact, we know that in the allosteric process the perturbation starts from the sensor site and reaches the active site through preferential paths with great efficiency. Quantum dynamics is critical to make energy/signal transfer highly efficient in large systems. This is evident in the light-harvesting process and the quantum approach applied to allostery that has been modeled on this process.

Nevertheless, quantum coherences present opportunities to control these transfers in multicomponent assemblies and/or macromolecules because of the way they modify the random hopping mechanism. In realistic situations with disorder, it is unclear whether quantum corrections to classical random walks help “speed up” energy/signal transfer; what is certain is they modify the pathways traversed by excitation.

Here, we will show that the efficient propagation of the perturbation from the allosteric site to the active site can be explained by a quantum approach. In this article, we will provide examples of systems studied with both QM and CM and we will compare them precisely on the problem of preserving the coherence and efficiency of the movement of the perturbation within the macromolecule. Since an efficient movement of the perturbation is essential to the allosteric process, this allows us to state that in the allosteric phenomenon there are essential aspects that cannot be analyzed without applying a quantum approach. To our knowledge, while a quantum approach has been applied and considered essential in other transfer processes in macromolecules, like in light-harvesting, it is the first time that an important characteristic of allostery, this fundamental biological process, has been related to quantum effect. Therefore, QM is important in studying the dynamics of allostery.

## Models of Allosteric Phenomenon

There are many models proposed to study this phenomenon. It is beyond the purpose of this paper to exhaustively analyze all these models. Here, we will only deal with some of these models that we intend to compare.

In the 1960s, Monod, Wyman, and Changeux (MWC) (Monod et al., 1965) proposed a simple model of allosteric interactions between two sites due to protein conformational changes. According to MWC, two coupled conformational states (named tensed *T* and relaxed *R*) of the protein co-exist in the thermal equilibrium. An allosteric effect is produced by the binding of a ligand in the allosteric site that changes the quaternary structure of the protein and generates an alteration of its active site. Since *R* state has a higher affinity for a second ligand in the active site than *T* state, the transition from *T* to *R* state, induced by the allosteric ligand, gives a modification of the affinity for the ligand in the active site. The MWC model requires a clear distinction among different conformational states and a different affinity for the ligand in the active site of these states. A key statement in the MWC theory is that the conformational equilibrium between these states is an intrinsic property of the protein and the role of the allosteric ligand is to stabilize the conformational state with a higher affinity for the ligand in the active site. Koshland, Nemethly, and Filmer (Koshland et al., 1966) (KNF model) proposed a slightly different model of the allosteric process with a sequential induced-fit of smaller conformational changes. In the KNF model, the ligand, binding to one of the subunits (allosteric site), causes a change in the structure of that subunit and produces an “induced-fit” change that starts from the neighboring sites and arrives to the active site.

The primary differentiating feature between the MWC and KNF models lies in the scale of conformational changes. While both suggest that the protein affinity for a given ligand changes upon the binding of the allosteric ligand, the MWC model suggests that this occurs because of a quaternary conformational change that involves the entire protein (moving from the global *T* state to *R* state). On the other hand, the KNF model suggests that these conformational changes occur on the level of tertiary structure (Ronda et al., 2013) within the protein. In this case, the neighboring subunits of the allosteric part change their conformation at the arrival of the first ligand and, consequently, this change propagates to the active site, with an increase of the affinity of this last site for a second ligand.

In addition to this kind of model, which assumed a large conformational change in the protein, there are other models with no structural changes, starting from that of Cooper’s and Dryden’s (Cooper and Dryden, 1984). In this last model, *R* and *T* states are structurally similar, and the difference between them appears in the fluctuations of the active site after the allosteric ligand binding. This last model can explain (a) allostery in the absence of pathways of structural distortions, (b) allostery in the absence of any structural change, and (c) the ability of allosteric ligands to act as agonists under some circumstances and antagonists under others. The difference between the models with large conformational modifications (both MWC and KNF) and the models without this change lies in the entropy and enthalpy contribution of Gibbs free energy in the allosteric process. We want to remember that the fast, local rearrangements around the stable position of single monomers of the protein correspond to the entropic part of the process, while the enthalpic part is related to a global and relatively slow motion that provokes conformational changes in a larger part of the protein. According to this, only processes comprising non-negligible enthalpy contributions result in global conformational changes, whereas purely entropic processes occur with no appreciable conformational changes.

A more recent model of the allosteric process is named EAM (ensemble allostery model) (Hilser et al., 2012; Tsai and Nussinov, 2014; Choi et al., 2015; White et al., 2018). It provides a generalization of both MWC and KNF models, allowing the treatment of a set of different populated conformational states (Hilser et al., 2012). In EAM, allostery is a consequence of the dynamic couplings among different sites of the system that allow a series of structural deformations to propagate in the network. In fact, all sites of the network are coupled, and the problem thus becomes that of obtaining the structural or dynamic changes caused by the excess of energy resulting from the binding of the allosteric ligand. As we said, until now the theoretical work carried out to characterize conformational changes in allosteric proteins has been mainly based on the analysis of the trajectories obtained from molecular dynamics. After locating a defined node with a protein constituent (e.g., amino acid), for example at the residue center of mass, a problem arises in computing the edges. Each pair of nodes is connected by an edge whose length is inversely proportional to the degree of interdependence between their motions (Bradley et al., 2008; McClendon et al., 2009, 2012). Another approach to studying coupled motions at the time scales required for understanding protein functioning is by using normal-mode analysis (Tasumi et al., 1982; Go et al., 1983; Bahar and Rader, 2005; Ma, 2005; Skjaerven et al., 2009). This analysis has been widely employed for detecting allosteric sites (Sethi et al., 2009; Su et al., 2014) and mapping allosteric pathways. Of course, normal-modes analysis has often been performed with classical mechanics, as in MD simulations, while, as we have already stated, the main aim of this paper is to go beyond a classical mechanics approximation.

A graph-theoretical or network approach (Watts and Strogatz, 1998; Albert et al., 2000; Amaral et al., 2000) are considered to be a natural choice for identifying allosteric networks (Christen et al., 2005; Di Paola et al., 2013; Di Paola and Giuliani, 2015). The network approach models the macromolecule with nodes (or sites) and edges to which different physical meanings have been attributed in literature (Hu et al., 2013). In this approach there has been a substantial effort to describe the pathways linking the allosteric and the active sites in a protein after the binding of the allosteric ligand (Atilgan et al., 2012; Gasper et al., 2012; Allain et al., 2014; Proctor et al., 2015). In fact, the focal concept underlying the network approach is precisely the identification of these allosteric pathways. The allosteric ligand binding results in a propagating cascade of residue movement throughout the structure until the active site of the protein is reached. By measuring these movements, e.g., using MD simulations or normal-mode analysis, these correlated motions are mapped onto a graph with nodes representing residues (or domains) and edges representing weights of measured dynamic properties in the system.

In our model, we have described the macromolecule as a network of nodes (or sites) and edges and, starting from a perturbation generated in the allosteric (or sensor) site, we have studied with QM how it moves in the network and reaches the active site. In our paper, we are using the standard QM where the Hamiltonian matrix is built up with the kinetic energy of the site (T_i_) and the potential energy of the coupling between the sites (V_i,j_), the eigenstates are found, and the time-evolution operator is applied to follow the dynamics of the perturbation:

(1) $$H=\sum_{i=1,}^{N\,s\,i\,t\,e}T_{i}+\sum_{i<j}^{N\,s\,i\,t\,e}V_{i,j}$$

(2) $$W\,\left(t\right)=E\,x\,p\,\left(-\frac{i\,H\,t}{\hbar }\right)\,W\,\left(0\right)$$

where **H** is the Hamiltonian matrix and **W** is a vector with elements related to the population **P** (or the amount of perturbation) of each site, with |W_i_(t)|^2^ = P_i_(t). It is the vector **W(0)** that defines the perturbation (which site is populated at *t* = 0) and its evolution **W(t)** in our approach. Let us assume the following numbering of the sites: the first site is where the perturbation wave is in *t* = 0 (the allosteric site) and the last site is the active site of the system. This identification of both the allosteric site and the active site is the starting assumption to study the two sites’ propagation in the allosteric process. The identification of the initial state with the allosteric site does not pose any problem, while a few comments about the active site identification must be done. Its specific character is due to the fact that, once joined by the perturbation, it uses the excess energy to achieve a chemical process (active site reaction) that we are not considering here because it was taken for granted.

Here, we want to emphasize three of the steps of our model. Firstly, if we want to study a specific system with allosteric phenomenon, we must identify some pieces of our protein that can be modeled with sites. Secondly, we must note the sites we need to calculate their properties and couplings with other sites. Finally, we must choose whether the properties of the sites and their couplings are constant or are time dependent as a function of the propagation of the perturbation. In these three steps, some modeling and calculation approximations may be necessary. For example, reducing the dynamics of our system to that of a few coupled sites is undoubtedly an approximation that transforms our real system into a model system, but it is also the choice of a specific quantum mechanical method of calculation for the electronic and vibration states considered for each site may represent a further approximation. This can also be said for the other aspects.

To us, the sites of the system could represent either the single amino acid of a protein or a larger fragment, but, more correctly according to a modeling perspective, the sites represent a larger piece of the protein sufficiently isolated and unitary that it can be considered a site (a protein domain, for example). Protein domains have been defined in literature as a portion of a polypeptide sequence, often in a single segment, that assume a stable 3D structure (Wriggers et al., 2005) and they are the basic functional and structural modules of proteins (Chothia et al., 2003; Vogel et al., 2004; Bhattacharyya et al., 2006) [the majority of proteins are multi-domains (Ma et al., 2011)]. We can describe each site of the macromolecular network by one or more states, which defines its characteristics. These states can be purely electronic states or states with an electronic and a vibrational component (vibronic states). The edges, instead, represent the physical interactions among these sites. These interactions have been considered to be electrostatic and/or van der Waals interactions in literature (Veloso et al., 2007; Vijayabaskar and Vishveshwara, 2010). In our modeling study, we will not detail this kind of interaction. In our model, each edge is a number that measures the coupling between the states of the nodes. We can consider the numerical value of the coupling between two sites constant or time-dependent and, in this last case, it can change in two different ways: (a) randomly or (b) as a function of the fraction of the perturbation wave present in the site. Following our time-dependent model of the macromolecules, we would like to describe proteins as dynamic entities where their folded structures are mainly consolidated by non-covalent intra-molecular interactions that can break and reform, providing these macromolecules with a high degree of flexibility and plasticity.

A general expression of the Hamiltonian of an allosteric process modeled with N sites, each one with an electronic state and M vibrational modes, can be the following:

$$\hat{H}=\sum_{j}^{N}\left(E_{j}\,|s_{j}>\,<s_{j}|+\right)+\sum_{j=1}^{N}\sum_{k\neq j}^{N}V_{j,k}\,|s_{j}>\,<s_{k}|+$$

(3) $$+\sum_{j=1}^{N}\sum_{l=1}^{M}\omega_{l}\,g_{j,l}\,\left({\hat{b}}_{l}+{\hat{b}}_{l}^{+}\right)\,|s_{j}>\,<s_{j}|+\sum_{l=1}^{M}\omega_{l}\,\left({\hat{b}}_{l}\,{\hat{b}}_{l}^{+}+\frac{1}{2}\right)$$

where |*s*_*j*_> is the electronic molecular state of the jth site with energy E_j_, V_j,k_ represents the coupling between the site jth and the kth site; ω*_*l*_* is the frequency of the nth mode constituting all the bath degrees of freedom of a site; *g*_*j,l*_ is the vibronic coupling on the jth site, and *b*^+^*_*n*_* and *b*_*n*_ are raising and lowering operators of the nth mode. In the expression (3), given the standard approximations of the sites, it is reasonable to assume that the displacement of the bath degrees of freedom remain small enough to be governed by almost linear restoring forces. Under this condition, the Hamiltonian bath can be approximated as a set of harmonic oscillators, and the exciton-bath interaction terms can be assumed to be linear in the bath coordinates. This approximation ignores the Duschinsky rotation effect, which can be significant if electronic transition causes non-trivial structural change of sites. Hence, while electronic-bath interaction terms may generally involve both diagonal and off-diagonal terms of the electronic Hamiltonian of the site, most models of perturbation transfer have assumed the existence of only diagonal couplings.

In conclusion, proteins are an ensemble of coupled sites in our modeling, and the allosteric phenomenon can be studied by a time-dependent quantum approach by following the redistribution among the sites of a perturbation, located in a specific site at *t* = 0. A specific group of states (or a single state, if we have one state per site) characterizes a site and the couplings between these groups of states (or state to state) characterize an edge. Here, we would like to underline that QM can consider systems with a large amount of coupled states. Besides, in the case of only a few couplings per state, it is possible to take into consideration hundreds of thousands of states, with the help of the Lanczos procedure (Lanczos, 1950; Golub and Von Loan, 1987; Lami and Villani, 1990).

Generally, a Hamiltonian, such as (3), consists of a huge matrix that can be sparse if we apply a model which considers null vibrational state couplings of different sites, as here. A generic sparse matrix *H* of order *n* × *n* can be approximated (Lanczos procedure) with a much smaller tridiagonal matrix *T* of order *m* × *m*, which has eigenvalues in the same energy range of *H.* This allows the exact dynamics of *H* to follow on from the dynamics of *T* in a limited range of time, so that it is possible to study a large number of states dynamics, even hundreds of thousands.

We can follow the propagation of the perturbation wave in the network along the sites, but, in order to study the allosteric phenomenon, we have characterized the propagation of the perturbation wave into two types of parameters. The first type is related to the first arrival of the perturbation wave into the active site and it describes three properties of this wave in this site: (a) the characteristic time to reach the maximum in the target t_a_; (b) the amplitude of the perturbation wave A_a_ (the wave attenuation corresponds to 1-A_a_ for a wave normalized), and (c) its spectral broadening B_a_. These three parameters can be obtained for any site by fitting P_i_(t) with a generic Gaussian,

$$P_{i}\,\left(t\right)=c_{1}*E\,x\,p^{\left(-\frac{{\left(t-c_{2}\right)}^{2}}{c_{3}^{2}}\right)}$$

in the range [t_i_-c_4_, t_i_ + c_4_] (with c_1_, c_2_, c_3_, and c_4_ real numbers) with P_i_(t_i_ ± c_4_) = P_*i*_(t_*i*_)/100 ≅ 0.

These three parameters well describe a simple propagation of a perturbation wave; in the cases of a more complex dynamic within the perturbation, these parameters must be referred to the first peak of the perturbation wave in the active site. In this case too, in fact, it will always be possible to identify the perturbation wave arrival time, the amplitude, and the broadening in the active site.

The second type of parameter is related to the efficiency of the allosteric process at a much longer time than that characteristic of the perturbation wave and it is associated with the average distribution of the perturbation in the sites. We have defined, as in literature [for example in LH process (Sue et al., 2020)] and in our previous studies (Ferretti et al., 1998; Ferreti et al., 2000; Villani, 2008), the perturbation transfer efficiency to a particular target (active) site *|a*> in terms of the time-averaged population of this site:

(4) $$<P_{a}\,\left(t\right)\geq \frac{1}{\tau }\,\int_{0}^{\tau }P_{a}\,\left(t\right)\,dt$$

After a time much greater than t_*a*_, <P_*a*_ (t)> becomes independent of time:

(5) $$<P_{a}\geq \frac{1}{\tau }\,\int_{0}^{\tau }P_{a}\,\left(t\right)\,dt\;\tau \gg t_{a}$$

Our modeling of allostery is based on the main characteristics of the largely used models of literature (MWC, KNF, models without conformation changes, EAM, and the network approach), but with some important differences. Firstly, our model is the only one applying QM to study the dynamics of the allosteric signal among sites. Secondly, another relevant difference between our model and the current models studying this phenomenon is the attention to time-dependent aspects of allostery. Until now, in fact, all scientists have affirmed that the perturbation moves in the network, but no one has related the characteristics of allosteric systems to the arrival time in the active site and to the propagation efficiency until arrival at that site. Finally, for the first time, in our model some properties of allostery have been related to the characteristics of the systems (number of sites, energies, and couplings) by simple rules.

## Light-Harvesting in Photosynthetic Phenomenon as Compared to Allostery

An important common feature shared by most of photosynthetic organisms is that they capture photons in pigment molecules embedded in the protein environments of light-harvesting complexes (LHCs). The excitons created in such LHCs remain well protected despite being waged by environmental fluctuations mainly due to protein degrees of freedom, and they are delivered successfully to their far away destinations in about hundred picosecond time scales.

A fundamental feature shared by many LHCs, which has been clarified during the past decade, is the intermediate nature of the terms constituting the Hamiltonian. In other words, for LHCs, it is common that the difference of energies between the states and the coupling terms constituting the Hamiltonian are of comparable magnitudes. Moreover, typically, these parameters are in the same order as those of the room temperature thermal energy and also of the major portion of the energy spectrum that comprises the macromolecule. The energetic convergence of these multiple terms provides LHCs with a rich repertoire of pathways and mechanisms for exciton dynamics and energy harvesting. At the same time, the lack of apparent small parameters in these systems render simple perturbation theories rather unreliable quantitatively. Therefore, advanced levels of quantum dynamics theories and computational approaches have become necessary to achieve accurate descriptions of exciton dynamics and relevant spectroscopic observables. In this sense, the excitons in LHCs have served as prominent testing cases for modern quantum dynamics, electronic structure calculation approaches, and spectroscopic methods.

All these characteristics are common to allosteric processes. In allosteric processes the energetic terms are often similar to each other and similar to those characterizing the interaction between the system and the environment, both in the case of degrees of freedom of proteins different from those of the two sites involved in the process, and for the environment surrounding the protein. However, in the allosteric process, although all those similarities are present, it is necessary that the perturbation leads the reaction site effectively. These similarities make it possible to apply the same methods to both processes.

There have been studies into three major LHCs: Fenna-Matthews-Olson (FMO) complexes of green sulfur bacteria, light-harvesting 2 (LH2) complexes of purple bacteria, and phycobiliproteins (PBPs) of cryptophyte algae (Jang and Mennucci, 2018). Here we will consider mainly the FMO complex.

The FMO complex is of great historical significance because it was the first LHC for which X-ray crystallography structural data became available (Fenna and Matthews, 1975; Fenna et al., 1977) and it has been subject to extensive spectroscopic and computational studies since then. The FMO complex can be expressed through eight sites (the eight bacteriochlorophyll-a molecules) and the zeroth order exciton Hamiltonian of this system with eight states. Earlier efforts to interrogate the energetics and the dynamics of excitons in LHCs have employed conventional subensemble non-linear spectroscopic techniques (Mukamel, 1995). During the 1990s, high-quality steady state linear spectroscopic data for FMO complexes, such as absorption, linear dichroism (LD), and circular dichroism (CD), became available (Miller et al., 1994; Savikhin and Struve, 1996; Louwe et al., 1997; Vulto et al., 1998; Wendling et al., 2002). Earlier efforts (Louwe et al., 1997; Vulto et al., 1998; Wendling et al., 2002) to explain this spectroscopic data were based on simple exciton models taking the form of eight sites, each one with an electronic state, without including bath interactions. Thus, line broadening due to environmental relaxation of exciton states and inter-exciton dynamics were not properly taken into account in these works. Nonetheless, the fittings of spectral data and the resulting model parameters turned out to be reasonable. This indicates that the exciton-bath coupling and inter-exciton couplings in the FMO complex are not dominant factors. Advances in the 2DES (2-dimensional electronic spectroscopy) technique made it possible to access new information on exciton states of the FMO complex that had not been available otherwise. Earlier success was made in determining detailed excitonic pathways (Cho et al., 2005; Bricker and Lo, 2015) which were largely consistent with a model exciton Hamiltonian developed earlier. This was soon followed by direct observation of beating signals lasting more than 500 fs at 77 K (Engel et al., 2007). Engel and coworkers made further progress, and they reported more detailed experimental results including the evidence that beating signals can be observed even at room temperature (Panitchayangkoon et al., 2010).

As for the FMO complex, a real time coherent beating signal was observed in 2DES data of LH2 complex. Angle resolved coherent four wave mixing spectroscopy showed evidence of coherent dynamics that can be separated from the relaxation signal due to the energy transfer (Mercer et al., 2009). Single molecule femtosecond pump probe spectroscopy, employing ultrafast phase coherent excitation, was also reported recently (Hildner et al., 2013). In the case of PBPs of cryptophyte algae, oscillations lasting up to about to 1 ps were observed, which were attributed mostly to vibrational coherences (Doust et al., 2004). Transient absorption spectroscopy (Doust et al., 2005) also revealed broad time scales of energy transfer within the PE545 ranging from 250 fs to picoseconds. A comprehensive set of absorption, CD, fluorescence, and time resolved transient absorption at both 77 K and 300 K were reported (Novoderezhkin et al., 2010).

In a theoretical study on these systems, it is possible to employ the standard quantum mechanical procedure to derive an exciton-bath Hamiltonian based on the assumption that the ground electronic state and the site excitation state can be defined in terms of direct products of adiabatic electronic states of independent chromophores defined at reference nuclear coordinates. The default choice for these reference nuclear coordinates is that of the optimized ground electronic states of chromophores. The common assumption, implicit in most theoretical models developed so far, is that the environment induced non-adiabatic coupling between the ground and the exciton state, is very small and hence negligible.

Quantum models used to study physical systems involved in light-harvesting consist of the same components needed to study the allosteric process. First of all, in both processes the complex system (the macromolecule) must be reduced to a small number of sites. This is possible using molecular mechanics studies which identify parts of molecules that are highly sensitive to the arrival of the perturbation into the allosteric site. Each site can be described electronically by one or more molecular states and the vibrational part can be added on each site specifically if relevant experiment information is known. The same applies to site couplings at both electronic and vibronic level. The only apparent difference between allostery and light-harvesting in photosynthesis is quantum coherences, which are not evident in allostery while they are present and studied in the second process. According to us, this does not mean that quantum aspects are not important in allostery, rather that it is not easy to prove them experimentally. In both processes, in fact, it is hard to believe that a classical approach could be sufficient to describe the complex dynamic.

## Allostery in Sequential Coupling (SC) Systems

When we reduce a macromolecule to a set of efficient sites, all the sites will be coupled. Although this is generally true, some consideration must be taken for sequential site coupling models. Sequentiality is necessary in every process from an initial site until you get to a specific final site. If all sites are equally paired, in fact, a perturbation generated in a site redistributes equally.

In allostery, the perturbation selective passage from the allosteric site to its specific active site is necessary. Obviously, nothing can assure that there is only one path to connect the allosteric site to the active one. This is the reason why, in our work, the case studies illustrate more than one connection path.

Moreover, each system can be approximated by a sequential coupled system and the dynamics of the original system is well described by the sequential system dynamics in a limited time range. This is the principle behind techniques like Lanczos’s that, by repeatedly applying a Hamiltonian matrix on a start state (door-state), constructs a tridiagonal matrix in a perturbative way. Of course, in Lanczos’s approach, the diagonal and extra diagonal elements of this matrix are all different. However, the model case with equal diagonal elements, equal to zero energy, and with equal extra diagonal elements among adjacent sites, is interesting. In this case, in fact, among equal diagonal elements the conservation of energy only selects degenerate sites vibronic states at the door-state and only a medium coupling is considered.

**FIGURE 1:**
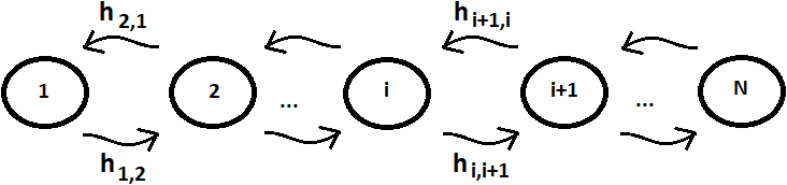
Schematic representation of a sequential coupled system.

Let us consider a system of N identical sequentially coupled sites (Figure 1) with T_i_ = cost and the coupling between the sites V_j,k_ of Eq. (3) equal: V_j,k_ = h_i,i+__1_ = h (this last approximation is not necessary, but it can be useful to simplify the analysis of the system) for any pair of neighboring sites. Since all sites are degenerate, we can assume the energy of the generic site to be |i> like the zero energy. The sign of the site coupling (h) is irrelevant in the case of N identical sequentially coupled sites. In QM, two degenerate sites |1>e |2> (E_1_ = E_2_ = 0) with couplings ± h, for example, produce two eigenstates of energy −h, + h, with a difference in energy Δ_e_ = 2 h that generate the same periodic dynamics (function of Exp [−i*Δ_e_*t]) between the states |1>e |2>.

This system is Markovian because the probability of transitioning between discrete sites is independent of previous transitions. This system can be studied in a CM approach by the help of its master equation of the process (for the details of this statistical study see example (Van Kampel, 1981):

(6) $${\dot{P}}_{i}\,\left(x\right)=h\,P_{i-1}\,\left(x\right)-2\,h\,P_{i}\,\left(x\right)+h\,P_{i+1}\,\left(x\right)$$

with ${\dot{P}}_{1\,\left(N\right)}\,\left(x\right)=h\,P_{2\,\left(N-1\right)}\,\left(x\right)-h\,P_{1\,\left(N\right)}\,\left(x\right)$ and *P*_1_(0) = 1*P*_*i*_(0) = 0*i* = 2,*N*

and where *P*_i_ is the population of site *i* and the amount of perturbation in the site *i*. The application of this master equation to this system gives, after a certain time, a uniform distribution of the perturbation in all sites since, following this classical approach, we are assuming that a perturbation spread is ongoing and it is redistributing the perturbation in all sites equally.

**FIGURE 2:**
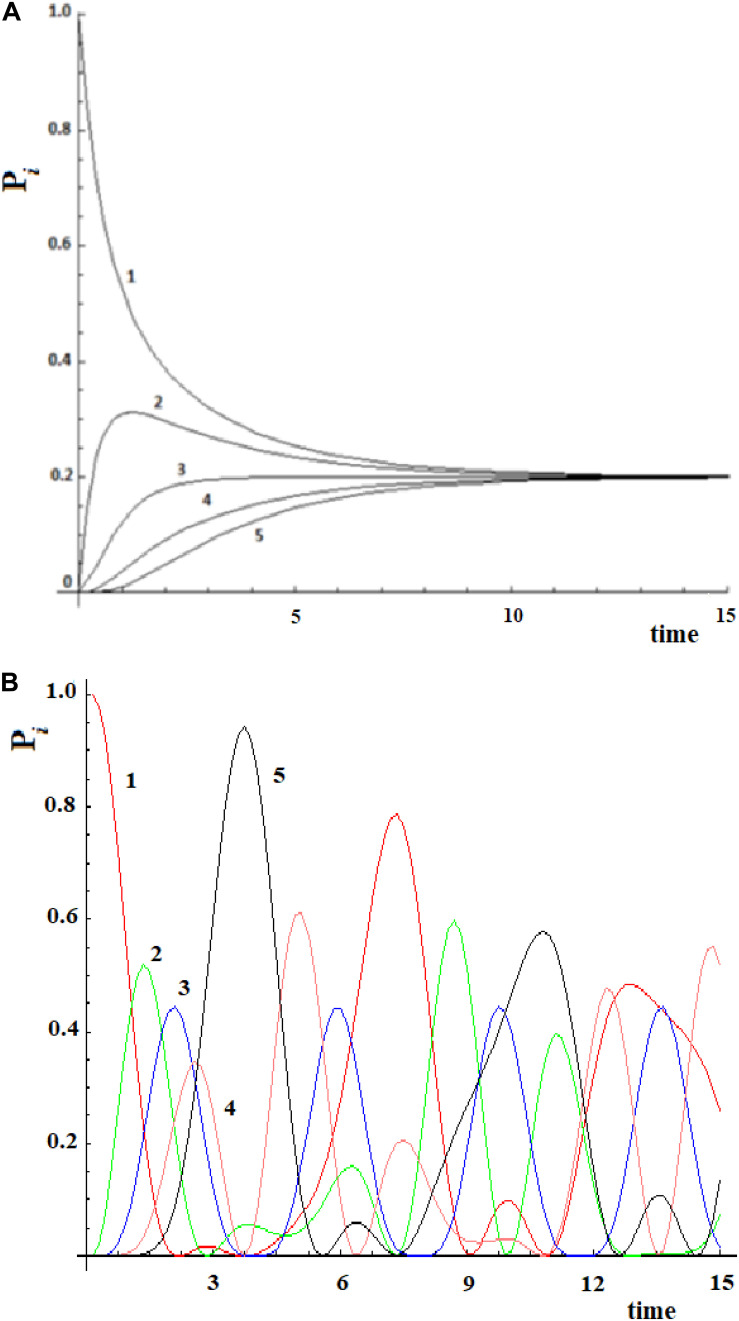
Time evolution of the site population, P_i_ with i = 1,5, in classical **(A)** and quantum **(B)** mechanics approach to five sites SC system. The numeration indicates the different P_i_ and, hence, the different fraction of wave perturbation in the site at different times.

Let us consider a simple case of five sites, where the perturbation at *t* = 0 in the initial allosteric site is |i≥|1> and with active site |a≥|5>. The time unit in this paper, unless otherwise noted, is the period 2*t_a_ of a degenerate states couple with coupling *h* = 1. In Figure 2A, this example studied by the master equation is shown and in Figure 2B we have reported the same case treated with standard QM. From the analysis of Figure 2A, it appears evident that CM does not encounter one of the essential characteristics of an allosteric phenomenon, which is that the active site is reached before the complete scattering of the signal. A quantum mechanical approach, instead, shows a movement of the perturbation along the sites and the arrival of the perturbation to the active site, as shown in Figure 2B.

Over the past few years, the application of a master equation has increasingly been used after the transformation of a protein in a set of sites and edges by MD simulations, suggesting that it is a promising tools to study allostery in a drug-discovery context (Pande et al., 2010; Chodera and Noé, 2014; Malmstrom et al., 2014; Schwantes et al., 2014; Bowman et al., 2015; Shukla et al., 2015). A detailed description of these applications to biological systems has been presented in two recent reviews (Chodera and Noe, 2014; Shukla et al., 2015). Here, we would like to consider only two examples in order to see the use of this Markovian model in the study of allostery.

Recently, Leitner et al. (2015) have examined vibrational energy flow in dehydrated and hydrated villin headpiece subdomain HP36 by master equation. The transition probabilities between the residues are obtained with an approach for computing frequency-resolved local energy diffusivities, which provide a map of communication between protein residues as a function of the vibrational frequencies of the modes that carry energy between them (Leitner, 2009). These authors have found that the results of the master equation compare well with all-atom simulations to about 1 ps following initial excitation of the protein, and quite well at longer intervals, though for some residues they observe deviations between the master equation and all-atom simulations at intermediate times from about 1–10 ps. In any case, after 10 ps both methods (analysis of master equations and all-atom simulations) for a system of N_site_ = 36 give a general result: the perturbation completely disperses in all residues with a population of 1/N_site_ (in this case 1/36 = 0.028) for each site. We have found a similar behavior in Figure 2A. With the master equation of the Markovian approach and with an MD simulation it is not easy to understand what allostery means since it is not possible to identify an active site preferentially connected to its allosteric site as well as finding a final state efficiently populated.

**FIGURE 3:**
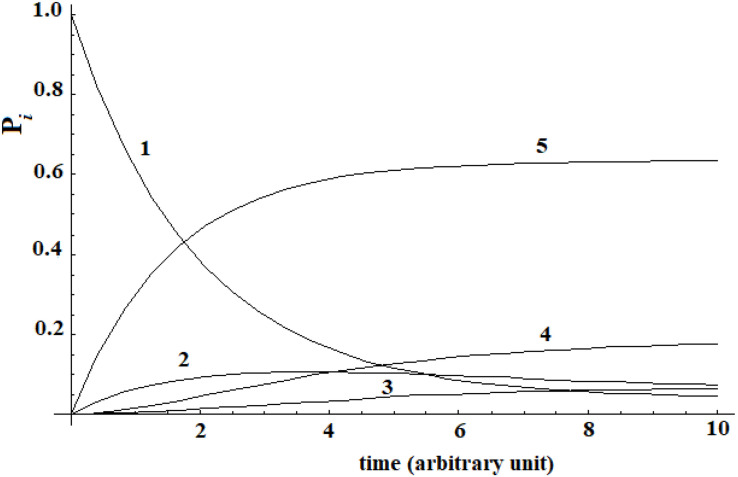
Kinetic of five-states model of Buch et al. (2011) that we have computed.

A different analysis has been done in a second example. In a recent study, Buch et al. (2011) was able to reconstruct the binding process of the enzyme-inhibitor complex trypsin-benzamidine by performing a large number of MD simulations of this system. The binding paths obtained shown the kinetic pathway of the inhibitor diffusing from solvent (|i≥|1>) to the bound (|5≥|a>) active state passing through three metastable intermediate states |2>, |3>, and |4>. In the Supplementary Table S1 of Buch et al. (2011), these authors give the matrix of transition probabilities for this five-state model. The kinetic evolution of this system has been computed by inserting this data in the master equation (Figure 3).

A different behavior can be highlighted comparing this last case with Leitner’s. In this last case, a complete dispersion of the perturbation is not present. The difference in the behavior of these two systems is due to the transition probability matrix among the states. In this last case, this matrix is not symmetrical [see Supplementary Table S1 of Buch et al. (2011)], while in the case of Leitner (2009) it was. In any case, we want to underline that, when a substantial passage (in this case 60%) from the allosteric site to the active site (Buch et al., 2011) can be identified, it is not possible to identify a propagation of the perturbation in the approach with the master equation.

Going back to the comparison between the CM and QM approaches on our five-state SC system, we want to highlight a different behavior in the propagation of the perturbation in the quantum approach (Figure 2B):

(1) In the quantum treatment, we see that the signal propagates like a wave of perturbation that moves along the sites, reaching the active site (the fifth state, in Figure 2B) after a certain time. It is possible to describe the allosteric process by the arrival time (we identify it with the maximum of the Gaussian), the amplitude, and the broadening (at 2/3 of the height of the Gaussian) of this wave in the active site.
(2) We are generally interested in the characteristics of the perturbation wave in the active site. It is possible, therefore, to find characteristics of the perturbation wave in any site. Our model, hence, allows us to determine the perturbation path in the network.
(3) Although there is a sequential movement of the perturbation wave, at any instant, the wave is never completely in a site (excluded at the initial time, of course).
(4) The perturbation wave has a simple form (like a Gaussian in time), but the process cannot be described with a single Gaussian moving among the sites. In a Gaussian moving in a space coordinate; if the amplitude decreases, the width must increase in order to preserve the normalization of the wave. This is not the case of the perturbation wave in time in any site and, in particular, in the active site. Moreover, since this site is the last one considered, and therefore it is not possible for the wave to proceed further, the perturbation wave is accumulated in this site before being reflected. This explains the difference between the perturbation wave in the active site compared to the other sites. This behavior, however, can be a good representation of the physical phenomenon that the signal at the active site is displaying.
(5) The perturbation wave moves efficiently in the network (it reaches almost a unitary amplitude in the active site of Figure 2B) and this means that the perturbation has moved almost completely from the sensor site to the active site.

The three parameters of the active site (t_a_, A_a_, and B_a_) that describe the allosteric process in SC systems are related only to the number of sites present (*N*) and to the value of couplings between the sites (*h*). In the analytical case of two sites (expressing everything in the atomic unit), we will have

(7) $$t_{a}=\pi /\left(2*\left|h\right|\right)\,B_{a}=\pi *\left(\left(180-109.471\right)/180\right)*h^{2}\;A_{a}=1$$

with 109.471 the tetrahedral angle.

**FIGURE 4:**
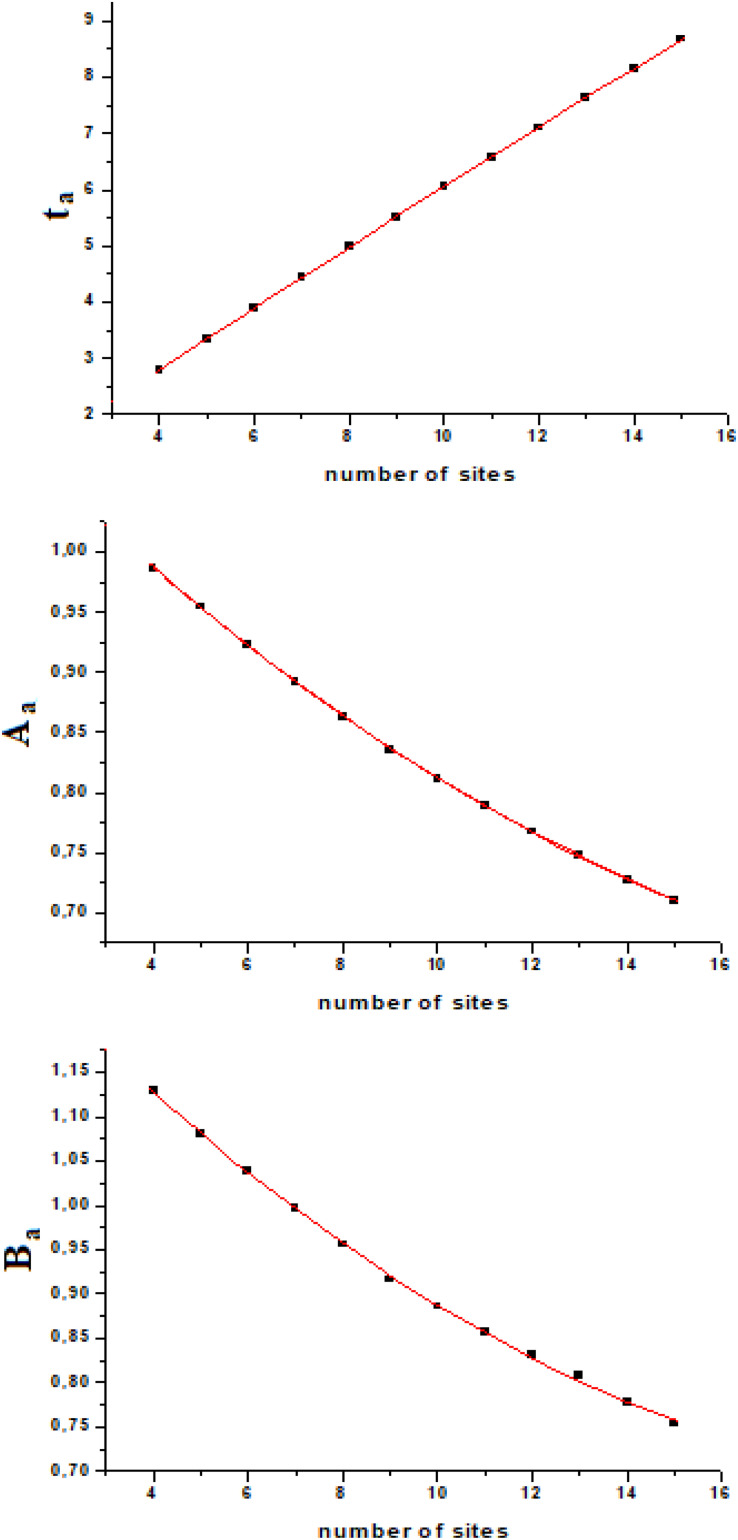
Fitting of the three parameters T_a_, A_a_, and B_a_ in the range (4–15) of number of sites.

If we calculate t_a_, A_a_, and B_a_ from the exact quantum dynamics of variable number of sites systems (we used number of sites from four to fifteen), we will find an almost linear relationship, as a function of the number of sites (Figure 4). Fitting this variation with a second order polynomial curve (in the interval of *N* from four to fifteen, [4–15]) with h_i,i+__1_ = 1, we will have

$$t_{a}=0.5595+0.5648\,N-1.5700*10^{-3}\,N^{2}$$

$$A_{a}=1.1368-4.0650*10^{-2}\,N+8.1642*10^{-4}\,N^{2}$$

(8) $$B_{a}=1.3379-5.7710*10^{-2}\,N+1.2700*10^{-3}\,N^{2}$$

with t_a_ and B_a_ which vary with the coupling as explained above (1/*h* and h^2^, respectively), while the amplitude of the perturbation wave in the active site, A_a_, is independent from the value of the coupling (when all couplings are equal, of course).

In this model system, we have obtained a result that we would like to highlight: the amplitude of the perturbation wave in the active site decreases (almost linearly in the range [4–15] of *N*) only as a function of the distance from the active site and the sensor one. From the analysis of Figure 4 it could be observed that the three parameters change almost in a linear way in a linear sequential system for a small number of sites (until 15) and that the time of arrival to the active site is the parameter that varies with linearity to a greater extent.

**FIGURE 5:**
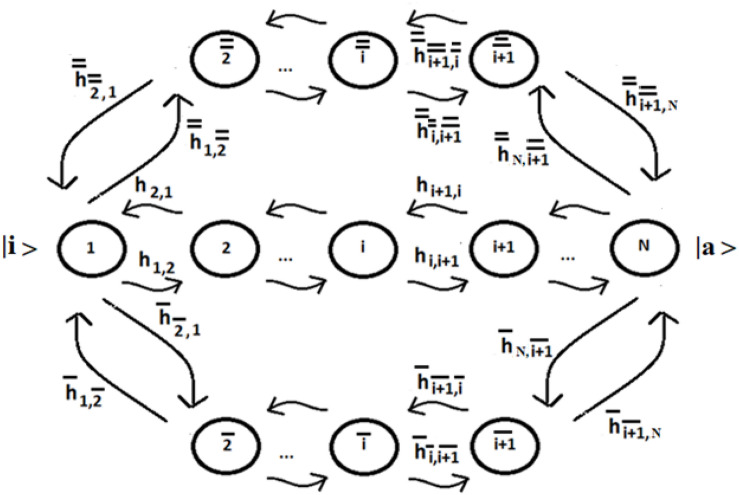
Schematic representation of three possible paths between the allosteric site |1≥|i>, populated at time *t* = 0, and the active site |N≥|a>.

This result can be used for more complex cases. Case studies where the initial (allosteric) site |i> has two or more alternative paths to get to the active site |a> are important and schematized in Figure 5.

**FIGURE 6:**
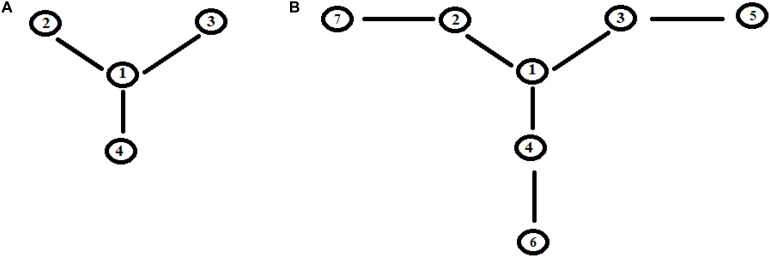
Three paths systems with 4 sites **(A)** and 7 sites **(B)**.

**FIGURE 7:**
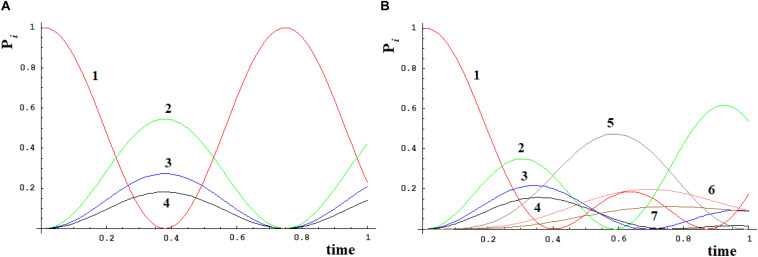
Time-dependent population of sites [systems **(A)** and **(B)**, Figure 6].

Let us apply a quantum approach in order to verify what happens when more than one path originates from a site. Let us examine two cases, **A**, **B**, as in Figure 6, where all sites have the same energy. In **A** from site |1> three different paths leave through the coupling h_1_,_2_, h_1_,_3_, and h_1_,_4_. In Figure 7A you find **A** (Figure 6) with h_1_,_2_ = 1, h_1_,_3_ = 1/√2, and h_1_,_4_ = 1/√3. It appears clear that the dynamics of the system has only one characteristic time and the populations in sites 2–4 will vary in function of h_i,j_^2^ and will be independent of the sign of **h** and of the sign of **c**, that relates to the coupling among the different ways (that is why parameter **c** is assumed as positive). In Figure 7B we have analyzed case **B** (Figure 6) with h_1_,_2_ = h_2_,_5_ = 1, h_1_,_3_ = h_3_,_6_ = 1/√2, and h_1_,_4_ = h_4_,_7_ = 1/√3. It is evident that the system dynamics are becoming even more complicated because there is more than one characteristic time despite the high symmetry of the system.

**FIGURE 8:**
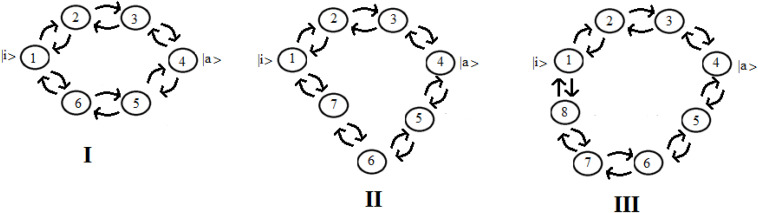
Schematic representation of two paths to get from site *|i*> to site *|a*> in the case of 6 **(I)**, 7 **(II)**, and 8 **(III)** sites.

Let us consider now three cases (Figure 8) that represent two paths to get from site *|i*> to site *|a*>. Case **I** represents two paths with the same number of intermediate sites; cases **II** and **III** are two paths of different lengths (with 1 and 2 sites more in a path). In particular, case **III** represents the approximation of the sequential coupling of the light-harvesting FMO complex.

Let us choose the path Pt_1_ = |1> → |2> → |3> → |4> as principal and study the modifications to this path adding the second path, Pt_2_, characterized by fewer couplings. If we decide to indicate the coupling between two sites with **h** (path Pt_1_) and c*h (path Pt_2_), same length (**case I**) or longer (**case II and III**), with **c** as a numerical factor between 0 and 1, for example in case **III** (one state per site) we will obtain a Hamiltonian:

(9) $$\text{H}=\left(\begin{array}{cccccccc} 0\; & \text{h}\; & 0\; & 0\; & 0\; & 0\; & 0\; & \text{ch}\\ \text{h}\; & 0\; & \text{h}\; & 0\; & 0\; & 0\; & 0\; & 0\\ 0\; & \text{h}\; & 0\; & \text{h}\; & 0\; & 0\; & 0\; & 0\\ 0\; & 0\; & \text{h}\; & 0\; & \text{ch}\; & 0\; & 0\; & 0\\ 0\; & 0\; & 0\; & \text{ch}\; & 0\; & \text{ch}\; & 0\; & 0\\ 0\; & 0\; & 0\; & 0\; & \text{ch}\; & 0\; & \text{ch}\; & 0\\ 0\; & 0\; & 0\; & 0\; & 0\; & \text{ch}\; & 0\; & \text{ch}\\ \text{ch}\; & 0\; & 0\; & 0\; & 0\; & 0\; & \text{ch}\; & 0 \end{array}\right)$$

**FIGURE 9:**
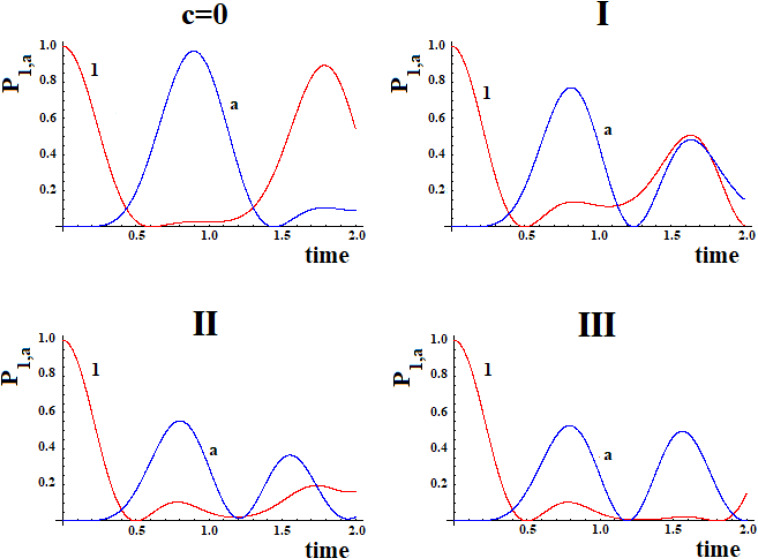
Population as a function of time of the allosteric site |1> and of the active site |a > for cases with c = 0 and **(I, II, III)** (Figure 8).

In Figure 9 we shown the population of site |1> and of site |a> in time with ***h*** = 1 for the case with *c* = 0 and for the three systems **I, II**, and **III** with ***c*** = 0.5.

The examination of Figure 9 shows that the initial site empties completely and the initial population fills the active site for a maximum fraction (A_a_) until about 80, 60, and 50% in the three cases. Obviously, the active site population, once reaching the maximum, decreases until 0, and then starts increasing again so that it can reach another maximum. Using as a reference the first arrival time of the perturbation to the active site, in the case of Pt_1_ (case with ***c*** = 0) the adding of a new path Pt_2_ alters slightly the arrival time of the perturbation to the active site and, generally, reduces it. Therefore, the t_a_ of the principal connection path between the allosteric site and the active site can be considered the maximum limit for multipath systems. This can also be applied to the enlargement of the Gaussian B_a_. Identifying the principal connection path between the allosteric site and the active site assumed a constant (medium) coupling between its sites, using Eq. (8) it is possible to estimate the three parameters t_a_, A_a_, and B_a_ of the complex system. This is an important achievement that deserves to be highlighted.

In order to evaluate the transfer efficiency from the initial site (allosteric) to the active one, we have to consider the values <P_a_(t)> in time, compared to the average population in other sites.

**FIGURE 10:**
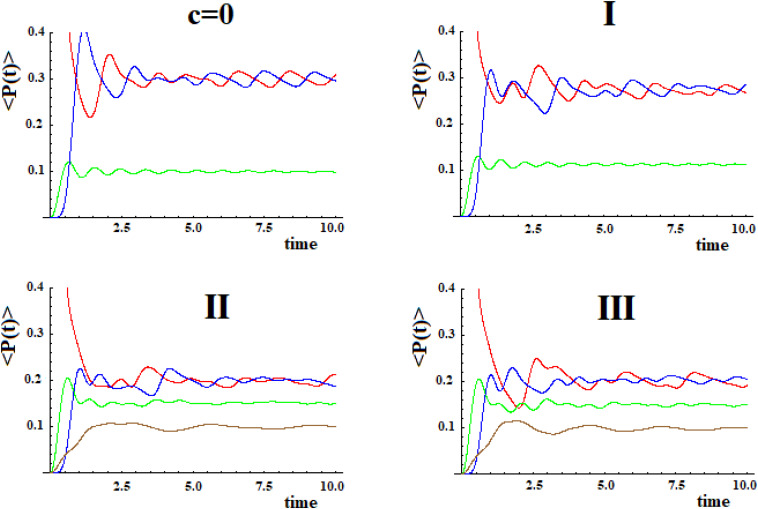
Average population of the sites depending on time (see text).

In Figure 10 shown in red <P_1_(t)> (state |i≥|1>), shown in blue <P_a_(t)> (state |a≥|4>), shown in green <P_j_(t)> (equal to (P_2_(t) + P_3_(t))/2 refers to the sites with coupling **h**), and shown in brown <P_k_(t)> (refers to the sites with coupling c*h) with P_k_(t) = P_j_(t) = (P_5_(t) + P_6_(t))/2 (case **I**; in this case, the brown curve is the same as the green one and it is not shown in figure), P_k_(t) = (P_5_(t) + P_6_(t) + P_7_(t))/3 (case **II**) and P_k_(t) = (P_5_(t) + P_6_(t) + P_7_(t) + P_8_(t))/4 (case **III**).

In Figure 10 (blue curve) the transfer efficiency (average population in long time, <P_a_>, compared to the first passage time in the site |a>) to the active site is between 20 and 30%. In particular, not all the perturbation has left the allosteric site (whose average population, <P_1_>, red curve, is the same as the active site) in long time, and the remaining perturbation, situated in different sites from the initial and the active ones (green and brown curves) is smaller than the one situated in the active site in all cases.

The prevalence of the active site compared to other sites depends on the quantum effect of coherent superposition between the perturbation wave arriving from a path and the wave arriving from the other path. In case **I** (assumed constant ***c*** = 1) this is evident. In this case, in fact, the two perturbation waves started from |i>, following the same paths, arrive at the same time and in phase in the state |a>. In Table 1, both A_j_, maximum amplitude in the site |j> at first passage and <*Pj*>, average population in the site |j> for long time compared to the first passage time have been reported.

**TABLE 1:** Parameters of the wave perturbation of Figure 10.

	**Site 1 (initial)**	**Site 2 = Site 6**	**Site 3 = Site 5**	**Site 4 (allosteric)**
A_j_	1	0.344	0.443	0.750
<P_j_>	0.278	0.111	0.111	0.278

In this case, the maximum population at first passage in the allosteric site is A_a_ = 0.750.

Despite the high symmetry of this system and its apparent simplicity there is no evidence that the perturbation moves completely from site |i> to the active site |a>. Also in this case, in fact, there are many different times (15 per N_sites_ = 6) that enter the time evolution in the form:

Exp[-i*Δ(E-jE)k*t],

  withj=1,2,..,Nesitesk=j+1,j+2,…,Nsites

as for case **B** (Figure 7).

In this system, the average population of the allosteric site for long time compared to the first passage time <P_a_> is 0.278. Please note that if there had been a complete diffusion of the perturbation, we would have obtained an average population equal to 1/6 = 0.167 in all sites.

## Sequential Coupling Systems With Variation of the Couplings Between the Sites at the Passage of the Perturbation (SC-PC) – a Non-Markovian Model

As mentioned before, there is a debate if the allosteric phenomenon has to be related to a conformational change or not and if this change is large (and related to the enthalpy term) or small (and related to the entropy term). In the previous paragraph, the perturbation does not change either the sites (energy) nor the edges (coupling) of the network. Here, we would like to test the idea that, during the movement of the perturbation along the network, the fraction of the perturbation wave present on the site changes the coupling between the sites. Of course, this is a non-Markovian process.

**FIGURE 11:**
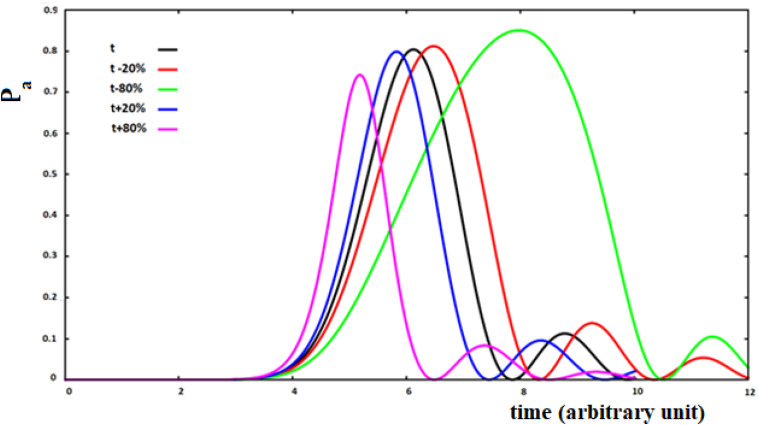
Population of the active site and perturbation wave in this site for 10 sites SC system. The value of the coupling is a function of the fraction of perturbation in the site (see text).

In Figure 11, we show the case of 10 sites sequentially coupled with h_i,i+__1_ = h between all pairs of sites adjacent to time *t* = 0. As a function of time, in this model we will have that:

(10) $$h\,{\left(t\right)}_{i,i+1}=\left(1+x*P\,{\left(t\right)}_{i}\right)*h$$

with *P (t)_*i*_* the perturbation fraction at site i and with the assumption of *x* = ±0.2, ±0.8 to study four different types of variations of the coupling between sites as a function of the perturbation fraction. We will use the following notation: *su* (small-up) for *x* = 0.2; *sd* (small-down) with *x* = −0.2; *lu* (large-up) with *x* = 0.8, and *ld* (large-down) with *x* = −0.8 for the four cases studied. The sign of the variation of the coupling between the sites describes a positive or a negative cooperation in the propagation of the perturbation wave. This means that the coupling between the two sites has been changed as a function of the fraction of perturbation wave at the starting site of the i → i + 1 transition, increasing or decreasing it because of what we want to describe.

Analyzing Figure 11, we can see that the increase of coupling between the sites, as a function of the perturbation, reduces the time of arrival to the active site, but in this case a wave of perturbation of smaller and narrower amplitude will arrive to the site and, vice versa, when the coupling decreases at the arrival of the perturbation. In any case, the effect of the decrease of coupling between the sites is greater than that of an increase of the coupling.

In order to connect the three parameters t_a_, A_a_, and B_a_ to the characteristics of the system, also for these non-Markovian cases, the fitting of these parameters was made according to the number of sites (from four to fifteen, *N* = [4–15]). In these cases, for the increase of coupling as a function of the perturbation, the precision of the fitting is similar to that of Figure 4. On the contrary, it is less precise in the case of a reduction of the coupling, i.e., the shape of the perturbation wave is less similar to a Gaussian (as seen in Figure 11 for the green curve). The results of the fitting of these three parameters as a function of the number of sites of the four cases studied (*su*, *sd*, *lu*, *ld*) are reported in Table 2.

**TABLE 2:** Parameters of the fitting of the four cases (*su*, *sd*, *lu*, *ld*) of Figure 11.

		**t_a_**	**A_a_**	**B_a_**
*su*	a1	0.44009	1.12985	1.78478
	a2	0.54593	–0.04158	–0.08125
	a3	–0.00123	8.69768*10^–4^	0.00186
*sd*	a1	0.72637	1.14744	0.96624
	a2	0.58371	–0.03990	–0.03766
	a3	–0.00193	7.71556*10^–4^	7.85480*10^–4^
*lu*	a1	0.23510	1.13055	3.50093
	a2	0.49429	–0.05116	–0.16357
	a3	−2.21174*10^–4^	0.00115	0.00366
*ld*	a1	2.06588	1.01862	0.15640
	a2	0.56941	–0.01826	0.01412
	a3	−4.75509*10^–4^	1.47453*10^–4^	−6.03616*10^–4^

(11) $$\mathrm{with}\;F=\mathrm{a1}+\mathrm{a2}*N+\mathrm{a3}*N{}^{2}$$

and F = t_a_, A_a_, B_a_.

We want to underline that, in the case of non-Markovian sequential coupling with a transition probability between two adjacent sites depending on perturbation (Eq. 10), the three parameters t_a_, A_a_, B_a_ vary in an almost linear way with the number of sites. Therefore, these three parameters can also be used to estimate those of a more complex system.

## Sequential Couplings Systems and With Random Environmental Variation of the Coupling (SC-RC)

Sequential coupling systems can be complicated in two ways to get closer to real situations. In the first case, we can add and/or remove randomly during the perturbation dynamics smaller couplings that bond spatially distant sites. This represents, for example, the case of a protein where some hydrogen bridges connecting distant sites have formed and then been destroyed. In the second case, it is possible to simulate a random interaction of the system with the environment that occurs while the perturbation wave moves inside the protein. This can randomly generate new non-sequential couplings (likewise the first case considered) or randomly modify the sequential couplings between the sites.

Physically, these modifications reflect the effect of the protein environment that dynamically modulates the site characteristics. All information about the system-bath interaction of each site is contained in its corresponding spectral density and in our model can be related to the reorganization energies of sites and/or the changes of couplings among them.

**FIGURE 12:**
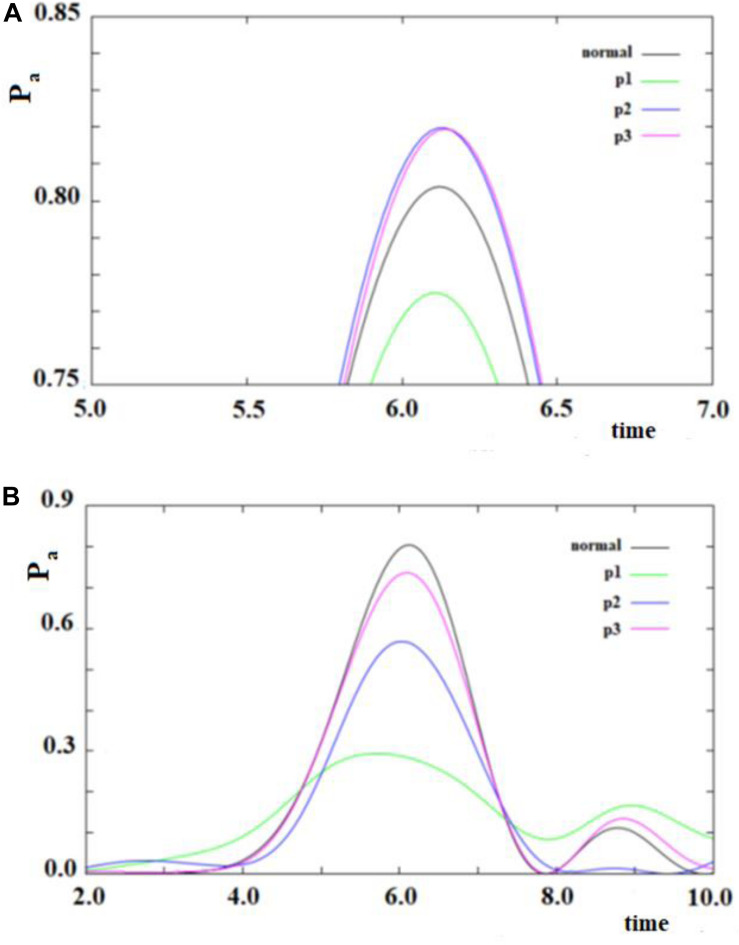
As in Figure 11, but with some (40%) values of the couplings changed randomly. Black curve: case without random perturbation; red, blue and green curves: cases with different frequencies of the random perturbation. (see text). **(A)** 0.2*h random variation of couplings, **(B)** 0.8*h random variation of couplings.

In Figure 12, we show the case of a SC system with 10 sites were, in addition to the non-perturbed case, there are three cases with random modifications of the coupling between the sites. In these cases, we have not distinguished a random creation of new couplings between the sites from a random variation of the sequential couplings. In particular, we have assumed a random perturbation of 40% of the couplings with a frequency of variation of 1/8 (p1), 1/4 (p2), and 1 (p3) time in each period of time of the system. In Figure 12A, the random amount of variation of the couplings is 20% of the sequential coupling, while in Figure 12B, the random amount of variation of the coupling is 0.8 time of the sequential coupling.

From the analysis of Figure 12, several things appear evident:

1. The system is largely resistant to a large random perturbation (40%) of the couplings. In fact, a perturbation of 20% of the amount of the sequential coupling in this case changes only a little both the arrival time and the broadening (result not reported) of the wave in the active site (Figure 12A). It is necessary that a very large perturbation (80%) of the amount of the sequential coupling occurs in order to considerably change this dynamic (Figure 12B). Physically, this means that the dynamics of SC systems are largely determined from the inside characteristics rather than from an environment perturbation during its dynamics.
2. A large random perturbation of the amount of the couplings leads to a reduction of the amplitude and to a larger broadening of the signal arriving to the active site. These effects increase when the random perturbation is more frequent.
3. The time to reach the active site is not very sensitive even to large random perturbations.

## A Model of State Sets Coupled in a Sequential Way

This model of the allosteric effect brings together the states of the system in different groups and then the groups of states are coupled. A set of states can be grouped in different ways, but in QM no specific difficulties arise if, with a model in mind, we compute the coupling of the states inside the groups and among the groups. For example, we can form a group of states with the vibrational states of each electronic state (vibronic states). In this case, it is possible to consider the electronic and the vibrational characteristics of allostery and also discriminate between energetic (E) and entropic (S) driven phenomenon in our approach. In a quantum oscillator, in fact, these two terms can be related to the partition function as follow:

(12) S=k⁢lnB⁡Q⁢(β)+E/T⁢E=-d/d⁢β⁢(ln⁡Q⁢(β))

with β = 1/(k_B_T) and Q(β) the partition function and k_B_ and T, Boltzmann constant and temperature, respectively. When the vibrational component of the vibronic states are equal to those of the harmonic oscillator of frequency ω, the partition function is:

(13) Q⁢(β)=Exp⁢(-β⁢h⁢ω/2)/(1-Exp⁢(-β⁢h⁢ω))

In a harmonic oscillator, the energetic term in the transition from a group of states to another can be associated with the change of equilibrium position, while the entropic term can be associated with the change of the frequency of the oscillator from a site to another. The first term is inversely proportional to the square root of the mass of the system modeled in the site since it implies a transition of ± 1 quantum, while the entropic term is inversely proportional to the mass of site since it is associated with a transition of ± 2 quantum. This means that for the typical mass of a site (residue) of 25 k Dalton, the energetic term generates an allosteric phenomenon with a time smaller than approximately two orders of magnitude compared with the entropic term.

**FIGURE 13:**
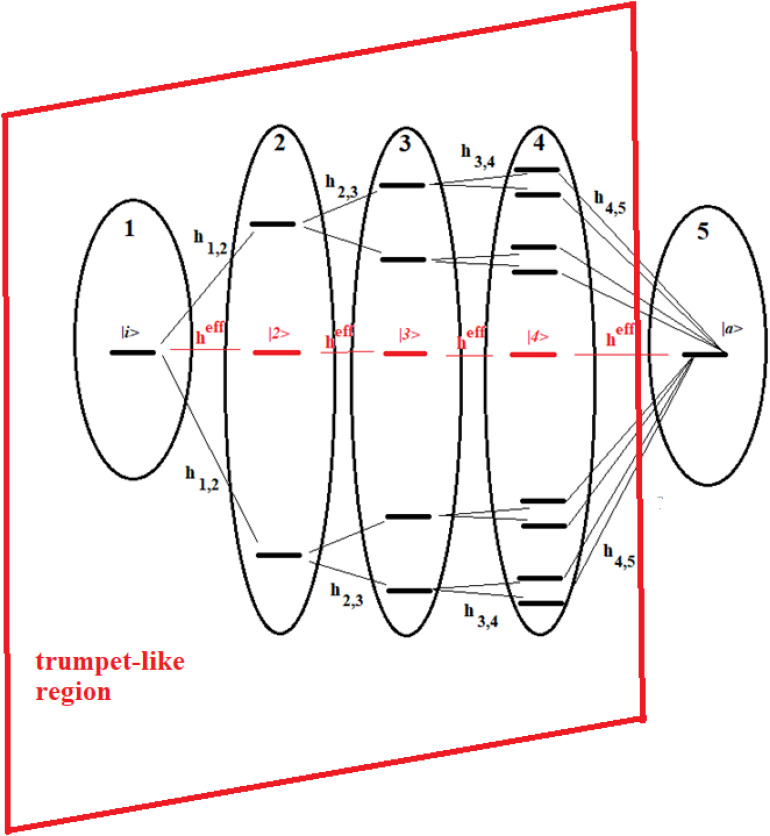
Schematic representation of a 16-site system divided into five groups of states. The effective state of each group is shown in red. Parts 1–4 form a trumpet-like region that connects the initial state (|i>) in the allosteric site to a region close to the active state |a>.

**FIGURE 14:**
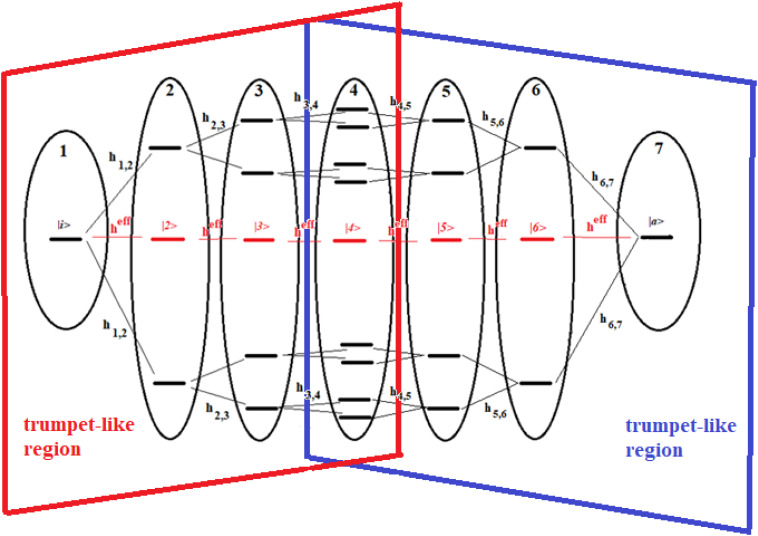
Schematic representation of a 22-site system divided into seven groups of states. The effective state of each group is shown in red. The double trumpet-like region is shown in figure.

The reason for considering groups of states and following their population over time is that, in this way, we can approximate complex systems with simpler ones, and we can study the dynamics of the former in an approximate way. Let us now consider the systems of Figures 13, 14.

These figures show a 16-site system and a 22-site system with non-sequential couplings, respectively. In these figures, the splitting and joining of states in the passage from a region to another does not take place in the energy field, but in the network field. In fact, they represent alternative paths in the network, but all the states considered have been taken at the same energy. Our goal is to study the dynamics of these two complex systems with that of two sequentially coupled systems of 5 and 7 degenerate states, those indicated in red in the figures (together with the states |i> and |a>).

The systems of Figures 13, 14 are two important models. The 16-site system of Figure 13 is formed by successive splits starting from the initial state, going from 1 to 2, 4, and 8 states. This group of states is then coupled to the state in the active site. This is what in literature is called a trumpet-system (Yan et al., 2017) because of its shape. In this system, in each step of splitting the states, the coupling is reduced and, therefore, the population of the relative state decreases. The system in Figure 14, on the other hand, can be considered a double trumpet-system where, after the splitting started from the initial allosteric state, there is a union of states up to the active one with a symmetrical increase of the couplings. The two systems considered here are simple and highly symmetrical models of the dispersion of the perturbation wave in the states of the network and its subsequent concentration, but our approach is also applicable to non-symmetric cases.

Recently, some authors (Yan et al., 2017) have used an elastic network to study the evolution of the perturbation along a trumpet-like constrained region that connects the allosteric site to the active one, and have found that the amplitude of the perturbation varies, not monotonically along the trumpet, even if the couplings between sites decrease monotonically from the allosteric site to the active one. In particular, they have found that the specificity of perturbation propagation is essentially controlled by the geometry of the network near the active site.

In order to compare the dynamics of the 16-site system and the 22-site system with those of the 5-site and the 7-site systems where,

h=i,jh=1

for all pairs of states |i> and |j>, the effective couplings between states of two adjacent regions in the 16-site system are assumed to be:

h=1,2eff√(h/2),h=2,3eff√(h/1,2eff2),

h=3,4eff√(h/2,3eff2),and h=4,5eff√(h/8)

and in the 22-site system:

h=1,2eff√(h/2)=h,6,7effh=2,3eff√(h/1,2eff2)=h,5,6eff

and h=3,4eff√(h/2,3eff2)=heff4,5

**FIGURE 15:**
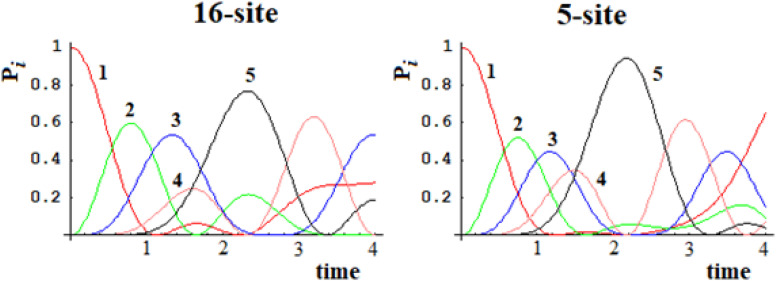
Population of the five groups of states of the 16-site system and of the 5-site system for comparison.

**FIGURE 16:**
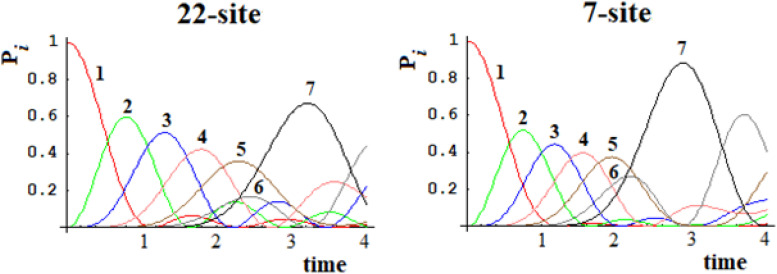
Population of the seven groups of states of the 22-site system and of the 7-site system for comparison.

In Figures 15, 16, we show the population of the five and seven groups of states of the 16-site system of Figure 13 and of the 22-site system of Figure 14, respectively, and the comparison with the 5-site system of Figure 2B and a 7-site system.

From Figures 15, 16, it is evident that both the dynamics of the 16-site system and that of the 22-site system are well approximated by the dynamics between the effective states of sequentially coupled regions. Obviously, this last temporal evolution of the perturbation wave is an approximation since the indirect couplings between all the sites have been neglected. With regard to the time and amplitude of the perturbation wave reaching the active site, however, these simplified studies are a very good approximation in both cases. This result was far from obvious since the search for an effective Hamiltonian is a widespread but very difficult quantum field of study.

**FIGURE 17:**
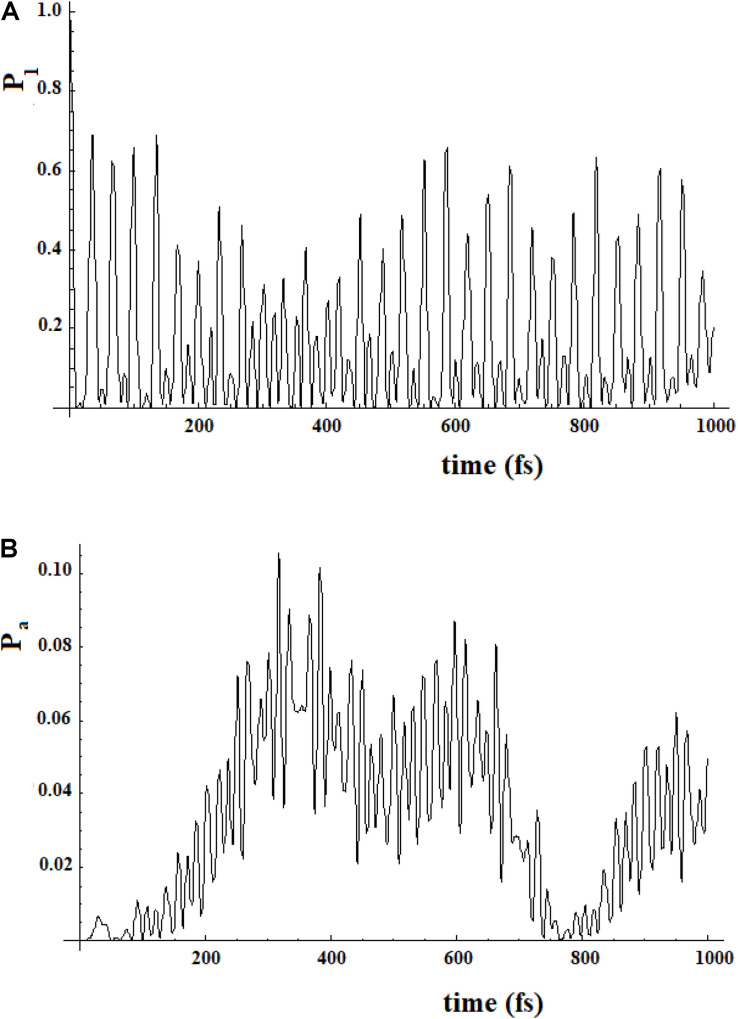
Population of the initial state |i≥|1> **(A)** and of the active site |a≥|20> **(B)** for a 20 sites SC system where the states are divided into four groups, each one of five states of equal energy. Each state of a group is coupled to all the states of the neighboring groups. The coupling between the groups is 10% (100 cm^–1^) of that inside the groups (1000 cm^–1^).

**FIGURE 18:**
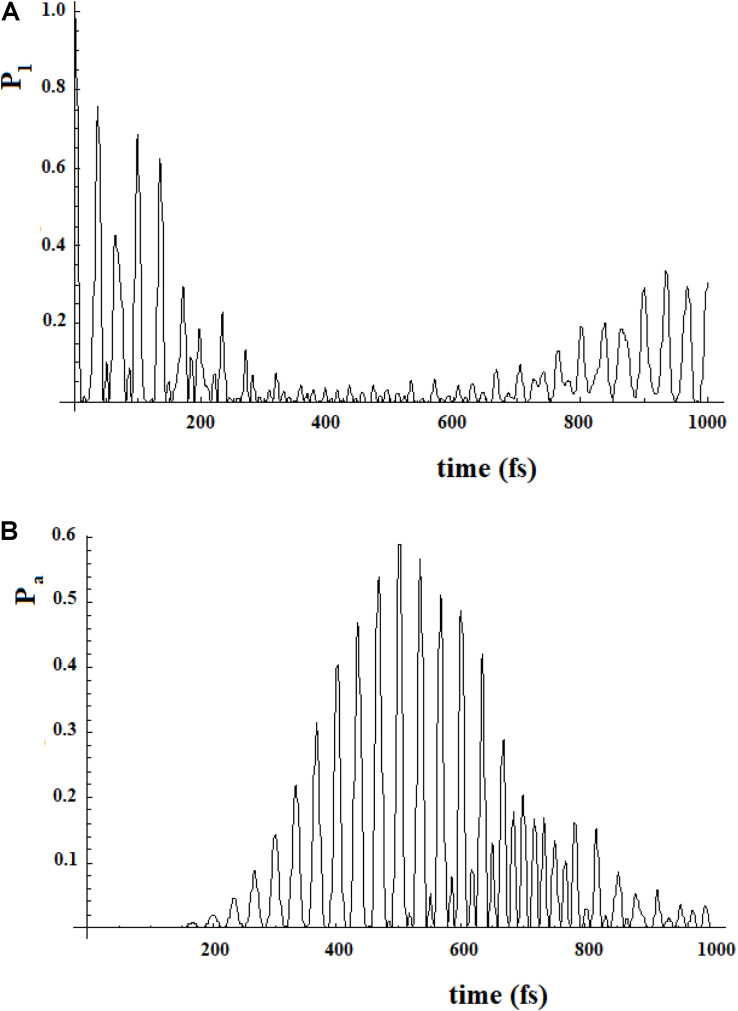
Population of the initial state |i≥|1> **(A)** and of the active site |a≥|20> **(B)** for a 20 sites SC system where the states are divided into four groups, each one of five states of equal energy. In this case each state of a group is coupled to all states of the neighboring groups. The coupling between the groups is 10% (100 cm^–1^) of that inside the groups (1000 cm^–1^).

To study a model system following this approach, here we have considered the case of a 20-state system divided into four groups of five states. Within a group, the coupling is sequential with *h* = 1, while among the groups, we have considered two cases (for both the coupling is 0.1*h): (a) each state of a group is coupled to all states of the neighboring groups; (b) only the last state of a group is coupled to the first state of the neighboring group. These two different types of coupling among the groups can be related to different models. In the first case, the ideal model is that of weakly interconnected groups; in the second, a sequential coupling in the groups is continued, from the last state of each group to the first state of the next group, with a weak coupling. Figure 17 refers to the first type of coupling and Figure 18 to the second type.

In Figures 17, 18, we can see that in both cases the time to reach the active site has lengthened considerably, compared to the SC system with 20 states. In both cases, there are two oscillation times. The difference between the two cases is in the percentage of the amplitude of the perturbation wave that reaches the active site. In the first case, in fact, it is small (about 10%) because the system is spread all over the states; in the second case, the percentage is higher (about 60%) because the system moves almost sequentially between the groups.

**FIGURE 19:**
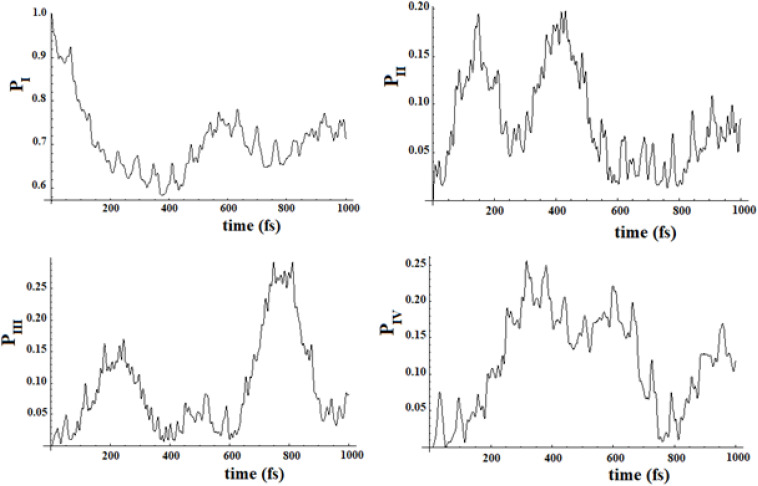
Population of the four groups of states of Figure 17.

**FIGURE 20:**
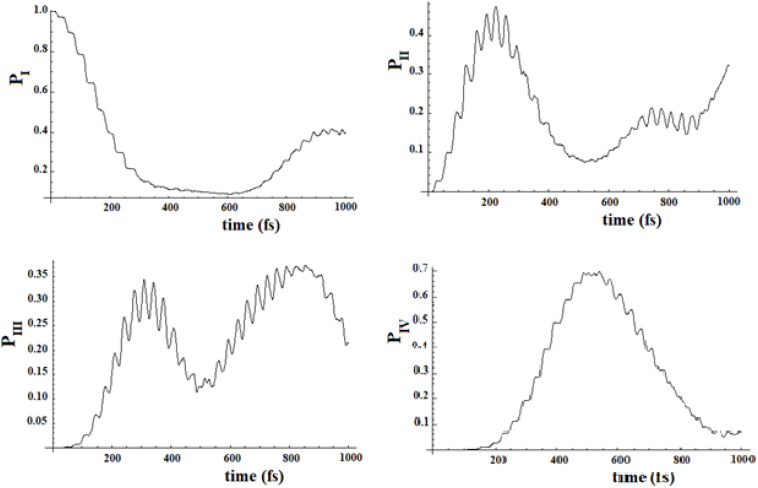
Population of the four groups of states of Figure 18.

In Figures 19, 20, we show the propagation of the perturbation wave in the four groups of states (in Roman numeration). It is evident that both the two oscillation times and the propagation of the perturbation wave form a group of states to the following.

## Conclusion

In this article, we have considered the allosteric phenomenon with a time-dependent quantum approach. To our knowledge, it has been the first time that such an approach has been applied to the study of the perturbation movement in the allosteric process. Quantum approach was used by Davidov to study the transport of energy by moving his soliton along the protein chains (Davydov, 1977; Georgiev and Glazebrook, 2019). Davidov’s soliton is a robust localized quantum quasiparticle representing a wave that propagates along the α-helix of proteins. The appearance of this quasiparticle is due to the coupling between the excitations of the amide of the peptide groups (excitons) and the phonons which correspond to a vibrational deformation. Our approach, however, is different from that of Davidov because it is not specific to a vibronic coupling and because we focused on how we can describe the perturbation, starting from the allosteric site, in the active site and on the mechanism that connects these two sites. In any case, there are also similarities between Davidov’s quantum approach and ours: both apply QM to protein processes, and both try to explain the persistence of the perturbation wave in highly complex systems.

In particular, we have described the allosteric process, considered as a propagation of the perturbation wave from the sensor to the active site, through three parameters of the first arrival in the active site: the arrival time, the amplitude and the broadening of this wave of perturbation in the active site, and through the transfer efficiency of the perturbation at a much large time than the time of first arrival.

Quantum mechanics has been widely applied to another fundamental macromolecular process: light-harvesting process in photosynthesis. The similarities between these two processes are evident: both require a site where the perturbation arrives and a second site (dozens of Ångströms away) where it produces an effect.

The long distance between the two sites and the presence of infinite degrees of freedom in a macromolecule make it necessary to explain the specificity of the propagation in both processes. It cannot be explained, in fact, by a master equation in terms of its spreading to all sites because, in this case, the process efficiency will be small. On the other hand, it is well known that the light-harvesting process is very efficient in transferring energy from the sensor site to the reaction site. Also in allostery we must disregard lower efficiency if we want to explain the specifics of a macromolecule site and understand that plays an allosteric function with regard to the active site.

Firstly, in this article we have compared our quantum approach to a stochastic one that uses a master equation for studying the evolution of the perturbation in the network of coupled states. In this case, we have analyzed the time-dependent evolution of the perturbation and we have shown that a quantum approach is suitable to follow the movement of the perturbation among the sites. The master equation, on the other hand, may have difficulties in reproducing the movement of the perturbation site-by-site or in identifying its possible paths in the network. Moreover, a master equation approach redistributes the perturbation in all sites equally, breaking the preferential relationship between the allosteric site and the active site. We then applied our approach to different types of model systems. In particular, we have studied four types of models:

(a) A case in which allosteric perturbation does not modify the couplings between the sites (coupled sequentially or with different paths, each one sequential coupled) during its movement in the macromolecule. This is a Markovian model.
(b) A second case in which the arrival of the perturbation modifies the couplings between the sites slightly or significantly, both increasing the couplings and reducing them. This is a non-Markovian model.
(c) Another case in which the environment changes the couplings between the sites randomly.
(d) A last case in which we grouped the states and then coupled these groups of states to each other.

In our study we attempted to draw general conclusions:

1. For a Markovian SC system, the perturbation wave moving from the start site to the arrival site is of a Gaussian type and the three Gaussian parameters (position, height, and width) are sufficient to describe the allosteric process of the first arrival to the active site. This is true both for linear SC systems and for multipath SC systems, estimated with a simpler case of the principal path. If the chemical process in the active site (not included in this description) is much slower compared to the first arrival time of the perturbation wave in this site, we must consider the average population of the active site, related to the efficiency of this allosteric process. We can link all these parameters to characteristics of the system (number of sites, energies and coupling).
2. When the coupling between the sites is time-dependent and related to the fraction of the perturbation wave that reaches a site, in this non-Markovian SC system the variation of the allosteric process depends both on the amount of the variation of the couplings and of its sign.
3. The system is resistant to a small random perturbation (that can modify sequential couplings or can add smaller couplings between different and not adjacent sites) during the propagation of the signal from the sensor to the active site. This means that the dynamics of this kind of system are mainly determined from the inside characteristics of the system itself than from the environment. A large random perturbation of the system alters the allosteric dynamics according to its frequency. The main characteristics changed are the amplitude and the broadening of the perturbation wave in the active site, while the arrival time is less sensitive. This random variation of coupling deserves further consideration. In our paper we have used the sequential coupling among sites with a constant value as a useful model to study more complex cases. The fact that a random perturbation, which modifies the coupling and/or creates new non-sequential couplings, does little to change the system dynamics tells us that our simple model can be used to predict the dynamics of more complex systems.
4. The allosteric effect treated with a model of sets of coupled sites states. In particular, we have considered two model systems, a 16-site system and a 22-site system, with the aim of studying their dynamics with the help of two sequentially coupled systems of five and seven states. With regard to the time and amplitude of the perturbation wave in the active site, we find that the simplified analyzes are very good approximations.

Furthermore, we studied two other model cases. If each state of a group is slightly coupled to all states of the neighboring group, the perturbation is distributed on all states of the system and the amplitude that arrives to the active site is small. On the other hand, if it is possible to think the neighboring groups of states coupled only by their final/initial state, the perturbation wave arrives into the active site with considerable amplitude. In both cases, the arrival times to the active site are significantly increased, compared to the SC case, and there are two characteristic periods of oscillation.

From this theoretical study of the main characteristics of the allosteric process, it is possible to draw some general remarks to deal with a real system that includes this process. First of all, it is necessary to identify the macromolecular parts which are involved in this process and that can be schematized as coupled sites. This can be done, as it is now, with molecular mechanics simulations. Once the sites are identified, their electronic and vibrational states and their couplings should be calculated with an accurate quantum calculation using a Born-Oppenheimer approximation and through spectroscopic techniques. We should then model the system with a set of coupled sites and find the preferential paths which lead from the allosteric site to the active one; finally, we should follow the dynamics using a quantum approach as shown in our work. We also point out that the quantum approach allows us to take into account the constructive interference among the different paths that connect the allosteric site to the active one. These quantum coherences can play a decisive role in complex systems with many paths. It is also not possible to consider the interference between the paths.

A last general question can be posed. If there is such a perturbation that moves along proteins in the allosteric process, is it possible to measure its speed of propagation experimentally? The answer seems to be positive. On the basis of recent pump-probe-type experiments, a conformational transition, similar to the one occurring upon ligand binding, can be found on a picosecond timescale by a laser pulse (Buchli et al., 2013). What is more, pump–probe MD simulations, where selected atoms are pumped by oscillating forces (Sharp and Skinner, 2006) and time-resolved femtosecond crystallography enabled by X-ray free-electron lasers (Tenboer et al., 2014; Barends et al., 2015) could shed light on important issues surrounding allosteric signal. In general, spectroscopic techniques constitute the most direct way of investigating energy transfer and link it to signal propagation in proteins. Direct determination of allosteric pathways through spectroscopic methods is therefore currently unfeasible, but we may gain indirect evidence for determining pathways of signal propagation by mutagenesis studies, where mutations at key residues deteriorate the efficiency of energy transport and lead to a loss of biological function (Kolodziej et al., 1996). Moreover, there is accumulating evidence to suggest that there are intra- and inter-molecular pathways of communication that help ensure the propagation of perturbations to distal sites and trigger the allosteric responses (Csermely et al., 2013; Di Paola et al., 2013; Papaleo, 2015; Papaleo et al., 2016). This propagation view corresponds well to the intuitive induced-fit description of allostery and it is supported by a sequence-based statistical method illustrating a connection between two sites through inferred allosteric networks (Suel et al., 2003; Hilser et al., 2012). We have proven the similarity between the allosteric process and the light-harvesting process in photosynthesis. Experimental techniques, which have highlighted light-harvesting quantum coherences, could be useful in studying the dynamics and eventually proving quantum coherences in allostery, too.

Finally, we want to return to the more general problem of a complex network of sites with interactions of different types (chemical bonds and weak interactions) among the components. These systems can form both macromolecules and less aggregated entities called supra-systems. In our opinion, the dynamic of these supra-systems should be studied with QM. Only a quantum approach allows us to understand the movements within this kind of weakly bonded network and allows us to study the preferential passage of a perturbation among their different sites. The idea that some biological processes, normally intended to function in a classical thermodynamic limit, may be able to use states and interferences of mechanical-quantum superposition is intriguing and challenging.

## Data Availability Statement

All datasets generated for this study are included in the article/supplementary material.

## Author Contributions

The author confirms being the sole contributor of this work and has approved it for publication.

## Conflict of Interest

The authors declare that the research was conducted in the absence of any commercial or financial relationships that could be construed as a potential conflict of interest.
